# Electrophysiological Signature of Emotional Processing in Participants with High Traits of Rumination During a Mood-Induction Task

**DOI:** 10.1192/j.eurpsy.2025.542

**Published:** 2025-08-26

**Authors:** C. Spironelli, Z. Romeo, F. Fusina, S. Messerotti Benvenuti, A. Angrilli

**Affiliations:** 1 Department of General Psychology, University of Padova; 2 Padova Neuroscience Center, University of Padova; 3 Neuroscience Institute, National Research Council (CNR), Padova, Italy

## Abstract

**Introduction:**

Past studies found lack of left frontal asymmetry in major depressive disorder (MDD) patients, also during task execution, probably depending on thought disorders associated to MDD. Indeed, individuals suffering from depressive mood are more likely to develop specific symptoms, i.e., rumination (R) (Thomsen, 2006) and perseverative thoughts (PT). According to Martin & Tesser (1996), rumination could be defined as *“[…] a class of conscious thoughts that revolve around a common instrumental theme and that recur in the absence of immediate environmental demands requiring the thoughts”.* Following this definition, this symptom can be represented along a continuum ranging between healthy individuals and patients with full-blown mood disorder.

**Objectives:**

The present study aimed at investigating the psychophysiological markers underlying the risk to develop mood disorders, in a community sample selected for two important psychiatric transdiagnostic domains, i.e., perseverative thoughts and ruminations (PT/R).

**Methods:**

In order to prompt a rumination state, we developed a new mood induction paradigm based on presentation of brief, validated videoclips able to evoke sadness, psychological sufferance and feelings of loss. Subjective reports and high-density EEG data from 20 students with high (≥80^th^) and 20 with low (≤20^th^ percentile) PT/R were collected. Subjective data analyses included self-perceived valence and arousal for ‘love abandonment’, ‘loneliness’ and ‘city documentary’ (i.e., neutral) clips. sLORETA source analyses on EEG bands were carried out to unmask the cortical areas involved in rumination.

**Results:**

Regardless of group, emotional clips elicited greater valence and arousal scores than neutral videos. Between-group sLORETA analysis revealed greater delta and theta activity in low vs. high PT/R participants in left superior parietal lobule during viewing of ‘love abandonment’ clips. Within-group analysis carried out in low PT/R participants showed that, compared with neutral clips, the ‘love abandonment’ and ‘loneliness’ conditions elicited greater alpha activity in superior (left) and middle (right) frontal gyri, respectively. On the contrary, high PT/R participants showed similar right (pre)cuneus alpha increase, regardless of emotional content.

**Conclusions:**

Results suggest a decreased sensitivity to negative videoclips in high PT/R individuals, together with the loss of the specialized frontal hemispheric valence-dependent asymmetry that is typically found in low PT/R participants.

**Disclosure of Interest:**

None Declared

